# Susceptibility results discrepancy analysis between NHSN Antibiotic Resistance (AR) Option and laboratory instrument data

**DOI:** 10.1017/ash.2022.185

**Published:** 2022-05-16

**Authors:** Youssoufou Ouedraogo, Christopher Evans, Daniel Muleta, Christopher Wilson

## Abstract

**Background:** The NHSN Antibiotic Resistance (AR) Option can serve as a useful tool for tracking antibiotic-resistant infections and can aid in the development of inpatient antibiograms. We recently described the frequency of antibiotic suppression in NHSN AR Option data. In this analysis, we describe the effects of suppression on practical uses of the NHSN AR Option, specifically selected agent antibiogram development, and detection of reportable conditions. **Methods:** Antibiotic susceptibility data were collected from the NHSN AR Option and commercial automated antimicrobial susceptibility testing instruments (cASTI) from 3 hospital networks. Data were obtained from January 1, 2017, to December 31, 2018. The clinical susceptibility data for third-generation cephalosporins and carbapenems against carbapenem-resistant Enterobacterales (CRE), *Pseudomonas aeruginosa*, and *Acinetobacter baumannii* were included. Susceptibility results were defined as suppressed when susceptibility results were observed from the laboratory instrument but not from NHSN data. For the overall percentage susceptibility estimation, isolates with <30 susceptibility results were excluded. Percentage susceptibility of NHSN results were compared to their counterparts from cASTI. **Results:** Of the 852 matched isolates in the primary analysis, 804 had at least 1 suppressed result. Of the 804 isolates, 16.9% were *P. aeruginosa*, 67.3% by *E. coli*, and 11.1% by *Klebsiella* spp. The following pathogen–drug combinations had no difference observed in the percentage susceptible between the 2 systems: ceftazidime tested against *P. aeruginosa*, ceftriaxone tested against *Klebsiella* spp, ertapenem tested against *Klebsiella* spp, imipenem tested against *E. coli* and *P. aeruginosa*, and meropenem tested against *P. aeruginosa*. Significant differences were observed for the following drugs tested against *E. coli*: ceftazidime (11.1%), cefotaxime (8.6%), and ceftriaxone (8.3%). In the NHSN AR Option, the following isolates showed suppressed results related to their phenotypic case definition: 17 (3%) CRE isolates, 7 (28%) carbapenem-resistant *Acinetobacter baumannii* (CRAB) isolates, 511 (93.2%) extended spectrum β-lactamase (ESBL) isolates, and 94 (66.7%) carbapenem-resistant *Pseudomonas aeruginosa* (CRPA) isolates. **Conclusions:** For select isolates, notably *E. coli*, we observed a large difference in the percentage of susceptible isolates reported into the NHSN AR Option compared to the cASTI data. This difference significantly limits the ability of the AR Option to create valid antibiograms for select pathogen–drug combinations. Moreover, significant numbers of CRAB, ESBL, and CRPA isolates would not be identified from NHSN AR Option because of suppression. This finding warrants the need for antimicrobial stewardship teams to regularly assess the impact of selective reporting in identifying pathogens of public health importance.

**Funding:** None

**Disclosures:** None

